# Effect of Reduced Versus Usual Lipid Emulsion Dosing on Bilirubin Neurotoxicity and Neurodevelopmental Impairment in Extremely Preterm Infants: Study Protocol for a Randomized Controlled Trial

**DOI:** 10.21203/rs.3.rs-2566352/v1

**Published:** 2023-03-07

**Authors:** Lindsay Holzapfel, Cody Arnold, Jon Tyson, Steven Shapiro, Eric Reynolds, Claudia Pedroza, Emily Stephens, Alan Kleinfeld, Andrew Huber, Matthew Rysavy, Amir Khan, Maria del Mar Romero Lopez

**Affiliations:** The University of Texas Health Science Center at Houston; Stanford University; The University of Texas Health Science Center at Houston; University of Kansas Medical Center; The University of Texas Health Science Center at Houston; The University of Texas Health Science Center at Houston; The University of Texas Health Science Center at Houston; Fluoresprobe Sciences (United States); Fluoresprobe Sciences (United States); The University of Texas Health Science Center at Houston; The University of Texas Health Science Center at Houston; The University of Texas Health Science Center at Houston

**Keywords:** bilirubin neurotoxicity, extremely preterm infants, lipid emulsions, randomized controlled trial, neurodevelopmental impairment, brainstem auditory evoked responses, phototherapy

## Abstract

**Background:**

Bilirubin neurotoxicity (**BN**) occurs in premature infants at lower total serum bilirubin levels than term infants and causes neurodevelopmental impairment. Usual dose lipid infusions in preterm infants may increase free fatty acids sufficiently to cause bilirubin displacement from albumin, increasing passage of unbound bilirubin (**UB**) into the brain leading to BN and neurodevelopmental impairment not reliably identifiable in infancy. These risks may be influenced by whether cycled or continuous phototherapy is used to control bilirubin levels.

**Objective:**

To assess differences in wave V latency measured by brainstem auditory evoked responses (**BAER**) at 34–36 weeks gestational age in infants born ≤750 g or <27 weeks’ gestational age randomized to receive usual or reduced dose lipid emulsion (half of the usual dose) irrespective of whether cycled or continuous phototherapy is administered.

**Methods:**

Pilot factorial randomized controlled trial (**RCT**) of lipid dosing (usual and reduced) with treatment groups balanced between cycled or continuous phototherapy assignment. Eligible infants are born at ≤750 g or <27 weeks’ gestational age enrolled in the NICHD Neonatal Research Network RCT of cycled or continuous phototherapy. Infants will randomize 1:1 to reduced or usual dose lipid assignment during the first 2 weeks after birth and stratified by phototherapy assignment. Free fatty acids and UB will be measured daily using a novel probe. BAER testing will be performed at 34–36 weeks postmenstrual age or prior to discharge. Blinded neurodevelopmental assessments will be performed at 22–26 months. Intention-to-treat analyses will be performed with generalized linear mixed models with lipid dose and phototherapy assignments as random effects covariates, and assessment for interactions. Bayesian analyses will be performed as a secondary analysis.

**Discussion:**

Pragmatic trials are needed to evaluate whether lipid emulsion dosing modifies the effect of phototherapy on BN. This factorial design presents a unique opportunity to evaluate both therapies and their interaction. This study aims to address basic controversial questions about the relationships between lipid administration, free fatty acids, UB, and BN. Findings suggesting a reduced lipid dose can diminish the risk of BN would support the need for a large multicenter RCT of reduced versus usual lipid dosing.

## Background/Rationale

Extremely premature infants have minimal caloric reserves. Because of the need to meet caloric requirements, early parenteral nutrition with carbohydrate, protein, and fat is recommended for preterm infants, particularly those born before 28 weeks’ gestational age (**GA**).(1) Soy-based lipid emulsion (e.g., Intralipid^®^), a commonly used parenteral fat preparation, is often initiated 1–2 days after birth. However, the optimal age and dose for administration is unclear. A recent Cochrane systematic review found no benefit of “early” administration of lipids (≤ 5 days after birth) versus later introduction.(2) The common initial lipid dose is 1 g/kg/d and advanced to 2 g/kg/d then to 3 g/kg/d on subsequent days with monitoring for tolerance; however no reports indicate that gradual increments in lipids improve tolerance.(3) The maximum lipid dose-related adverse effects including chronic lung disease, increase in pulmonary vascular resistance, impaired pulmonary gas diffusion, sepsis, free radical stress, and bilirubin neurotoxicity (**BN**).(3–5) Whether a relatively high caloric intake from parenteral lipid emulsions is better than a relatively low intake is also unclear.

Free fatty acids (**FFA**) compete with bilirubin for albumin binding sites. There is a long-standing concern that lipid emulsions may increase FFA sufficiently to displace bilirubin from albumin and increase serum unbound bilirubin (**UB**) that passes into the brain and causes BN.(6) This concern was heightened by a 2017 report where lipid doses of 2–3 g/kg/d were associated with an increase in unbound free fatty acids (**uFFA**) and UB among infants < 28 weeks’ GA.(7) Whether these increases resulted in BN or neurodevelopmental impairment (**NDI**) was not determined. Amin, Maisels, and Watchko, authorities on neonatal hyperbilirubinemia, concluded from the report that “current observations suggest that a more tempered approach to early lipid infusion in neonates ≤ 28 weeks’ GA should be considered, until we have a better understanding of the risks and benefits”.(8) Notably, UB levels are more closely related than total serum bilirubin (**TSB**) values to the risk of BN.(9) However, equipoise exists between whether peak UB peak, mean UB, or serial UB area under the curve is most predictive of NDI. In Hegyi et al,(7) phototherapy was found to reduce both TSB and UB in all infants, but when evaluating the infants < 28 weeks’ GA separately, TSB was reduced but UB was not, suggesting that UB may be a more preferred method to measure risk bilirubin toxicity. A persistent problem in evaluating the relationship of parenteral lipid emulsion and BN risk is the lack of a reliable, well-validated, and commercially available method to assess UB. Therefore, further investigation of UB as a measure of BN is warranted.

A sensitive measure of BN is via the brainstem auditory evoked response (**BAER**). BAERs are noninvasive, computer-averaged surface recordings of the electrical responses of conduction through fast conduction, synchronized firing of the afferent auditory nervous system from the auditory nerve through the brainstem pons and midbrain up to the lateral lemniscus entering the inferior colliculus in response to a click stimulus.(10) Conduction abnormalities due to BN can be reversible or permanent.(11–13) Typically the latencies (time from onset of the click to the peak of waves) are recorded for waves I (distal auditory nerve), III (cochlear nucleus), and V (lateral lemniscus in the midbrain entering the inferior colliculus, which is also highly sensitive to BN but doesn’t show up well on the BAER.(11) However, BAER wave V is often most prominent and reliably obtained and is the standard primary variable outcome measure for BN in neonates. In the largest pragmatic randomized controlled trial (**RCT**) of phototherapy dosing,(14) BAERs were performed at one site for infants 34–36 weeks post-menstrual age (**PMA**). For infants receiving cycled compared to continuous phototherapy, there were reduction in wave I, III, and V latencies for infants born at 751–1000 g, and non-significant similar trends in infants of born at 501–751 g with no differences in interwave intervals.(15) This suggests improved conduction at the distal auditory nerve with cycled phototherapy dosing.

BAERs provide a novel method to directly measure the potential effects of BN on neurological function from the reduction in the usual lipid dose on UB deposition, and UB is proven to predict abnormal BAERs in a diverse newborn population.(16) Irrespective of what lipid dose is administered, phototherapy is routinely used to reduce bilirubin levels and avoid BN in extremely low birthweight (**ELBW**) infants during the first 1–2 weeks after birth. Recent evidence suggests that cycled phototherapy has similar effectiveness with less photo-oxidative injury than with conventional continuous phototherapy for ELBW infants.(17–19) However, further titration of phototherapy dosing is needed for the < 750 g birthweight infants because aggressive phototherapy in comparison to conservative phototherapy had higher rates of death with a reduction in profound NDI in infants < 750 g birthweight with aggressive phototherapy but no difference when profound NDI and death were assessed as a composite.(14) For this reason, the National Institute of Child Health and Human Development (**NICHD**) Neonatal Research Network (**NRN**) is conducting a RCT to determine whether cycled versus continuous phototherapy reduces mortality for ELBW infants. Because our center is participating in that trial, we have the opportunity to assess whether reduced lipid dosing decreases the risk of BN below that of usual lipid dosing irrespective of whether cycled or continuous phototherapy is used.

## Objectives

The primary objective of this study is to conduct the largest feasible study of infants at high risk for BN to assess whether a reduced lipid dose will decrease the latency of BAER wave V relative to that of infants with the usual lipid dose, irrespective of whether cycled or continuous phototherapy is administered. Infants ≤ 750 g birthweight or < 27 weeks’ GA will be randomized to receive usual or reduced lipid dose, which is half that of the usual dose, during the first two weeks after birth. BAERs will be performed at 34–36 weeks’ PMA or as soon as feasible prior to discharge of the infants. This is the earliest PMA BAERs are reliably assessed in a neonatal intensive care unit (**NICU**). We hypothesize among infants ≤ 750 g birthweight or < 27 weeks’ GA, usual and reduced lipid dosing during the first two weeks after birth will result in similar BAER wave V latencies.

Secondary objectives will compare infants randomized to usual or reduced lipid dose groups for (1) bilirubin measures, (2) nutrition and growth parameters, (3) in-hospital outcomes and (4) NDI follow up assessments at 2 years.

We hypothesize infants receiving usual lipid dose compared to reduced lipid dose will have increased mean and peak UB levels, similar nutrition and growth parameters at all time points, and will have a greater need for mechanical ventilation at 5 days, and have similar NDI follow up assessments at 2 years. Additionally, we anticipate no evidence of an interaction between lipid and phototherapy groups.

## Methods/Design

This study protocol meets the Standard Protocol Items: Recommendations for Interventional Trials (**SPIRIT**) statement.(20)

This single center, pragmatic, parallel group, RCT compares infants to receive usual or reduced lipid dose, half the dose of usual lipid dosing, during the first two weeks after birth. All participants will also be enrolled in the NICHD NRN Cycled Phototherapy Trial (NCT03927833) (as approved by the NRN Concurrent Research Committee). Infants will be stratified before randomization to usual or reduced lipid dose by whether they were randomized in the NRN trial to receive cycled or continuous phototherapy.

### Study Setting and Context

Recruitment began on January 8, 2021, and is ongoing at the Children’s Memorial Hermann Hospital (**CMHH**) in Houston, Texas affiliated with the University of Texas Health Science Center at Houston at the McGovern Medical School. CMHH NICU is a 118-bed level-IV NICU with an average of 110 deliveries of preterm infants ≤ 1000 g birthweight yearly. Certified examiners perform neurodevelopmental assessments for > 90% of ELBW infant survivors at 22–26 months. CMHH is one of 15 current centers in the NICHD NRN. The CMHH NICU is participating in 7 active NIH-funded trials currently and has enrolled > 1000 patients since 2004 in all NRN trials since 2004.

### Study Population and Eligibility

Infants are screened for eligibility and enrolled within 36 hours after birth. Inclusion criteria include inborn infants at CMHH NICU < 27 weeks’ GA by best obstetric estimate or ≤ 750 g birthweight. Exclusion criteria include major congenital anomaly, overt nonbacterial infection, receiving pure soy-based lipid emulsion prior to enrollment, or declined consent for the NRN Cycled Phototherapy Trial.

### Consent

Patients are approached after informed consent is granted for the NRN Cycled Phototherapy Trial. Informed consent is obtained by a research team member in person in English or Spanish, or via phone interpreter in the caregiver’s primary language. The Institutional Review Board at McGovern Medical School approved this protocol (HSC-MS-20–0916) and consent form on October 13, 2020. Protocol amendments will be updated via ClinicalTrials.gov.

### Randomization and Allocation

**Figure 1:**
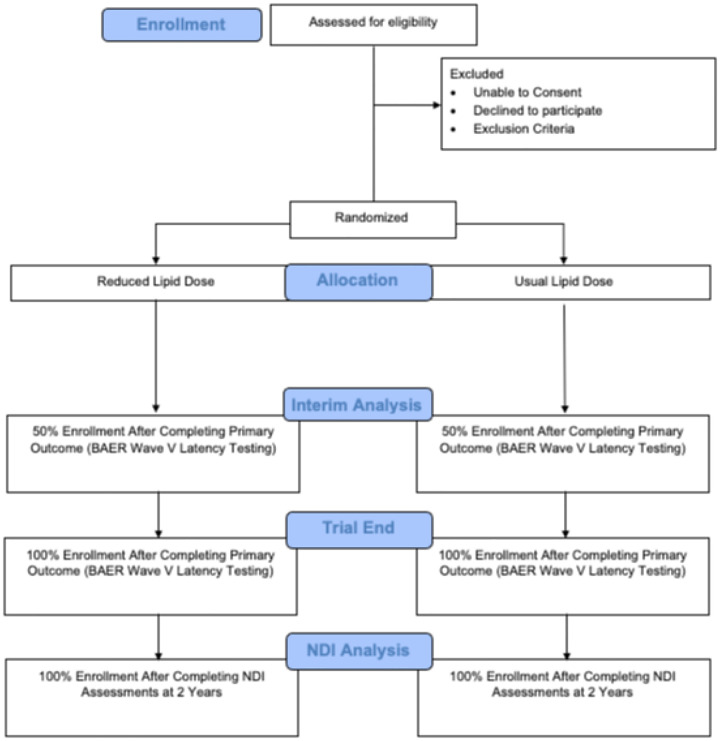
Patient Flow Diagram.

Infants for whom informed consent is obtained are stratified before randomization by the phototherapy treatment group they were assigned for the NRN trial so that half of each lipid group will receive continuous phototherapy and half will receive cycled phototherapy for TSB > 5 mg/dL (Fig. 1). The patients will then be randomized 1:1 with variable block size to either usual or reduced lipid dose using a web-based computerized program (REDCap).

### Blinding

Blinding to the lipid group assignment is not feasible for clinical personnel, parents, or the principal investigator (L.H.). The statistician (C.P.), laboratory co-investigator (A.K., A.H.), BAER expert co-investigator (S.S.) and data safety monitoring panel will remain blinded to lipid group assignment.

### Treatment Groups

Usual lipid dose group (Control): The lipid emulsion will be started at 1 g/kg/d and advanced by 1 g/kg/d each day to a max of 3 g/kg/d as per usual clinical practice (Table 1) in our center and supported by product information approved by the Food and Drug Administration.(21)

Reduced lipid dose group (Intervention): The lipid emulsion will be started at 0.5 g/kg/d and routinely advanced by 0.5 g/kg/d each day to a maximum of 1.5 g/kg/d (half the usual lipid dose) (Table 1). To prevent essential fatty acid deficiency, a minimum linoleic acid intake of 0.25 g/kg/d is needed in preterm infants, which is delivered in 0.5 g/kg/d dose of a pure soybean-based intravenous lipid emulsion.(22)

The assigned treatment group (usual or reduced lipid dose) will remain in effect for a minimum of 7 days and a maximum of 14 days when bilirubin levels are likely to be highest (Table 2). On days 10–14 after enrollment, the intravenous lipid emulsion type and dosage may be changed at the discretion of the clinical team if phototherapy is discontinued for ≥ 48 hours and the TSB < 5 mg/dL. Thereafter, the lipid preparation and dosage may be changed at the discretion of the clinical team. Throughout the study period, the infusion rates for both doses may be decreased, and advances may be delayed for lipid intolerance providing documented justification in the daily progress note. Lipid intolerance is determined by triglyceride levels > 250 mg/dL. Lipid emulsions will be routinely initiated within 48 hours after birth, as per routine clinical practice at CMHH NICU. The dates and times of start, stop, and changes in lipid infusion rates will be recorded. The PI will conduct daily monitoring of the parenteral nutrition orders for adherence to lipid dosing.

### Outcome Measures

The primary outcome is wave V latency measured by BAERs performed at 34–36 weeks’ PMA or before hospital discharge.

Secondary outcomes will include bilirubin measures, nutrition and growth parameters, hospital outcomes, and NDI follow-up assessments. Specifically, bilirubin measures in the first two weeks that will be included are mean UB concentration > 10.3 nM/L (0.6 mcg/dL) and > 13.7 nM (0.8 mcg/dL), proportion of UB measurements > 10.3 nM/L (0.6 mcg/dL) and > 13.7 nM (0.8 mcg/dL). These cuts offs were chosen based on unpublished data showing sharp increase in risk of severe NDI at the 85th percentile, or at 10.3 nM/L (0.6mcg/dL) among infants in the prior NRN trial (14), and the exchange threshold for 22–27 week GA infants in Japan (23) using an automated UB analyzer (Arrows Co, Ltd, Osaka, Japan),(24) which uses the glucose oxidase peroxidase method. Additional bilirubin measures will include, peak UB concentration, mean uFFA, peak uFFA, peak TSB, and proportion of direct bilirubin > 1.5 mg/dL before discharge. Detailed daily nutrition and growth parameters will be monitored in the first two weeks, at 21 and 28 days after birth, 34 and 36 weeks’ PMA, and discharge at 2 years. At these time points the macronutrients prescribed consisting of parenteral and enteral protein, fat and carbohydrate doses in g/kg/d, weight velocity (g/kg/d) and sex-specific Z scores changes (25) from birth through day 28 and at 36 weeks’ PMA will be measured. Hospital outcomes will include serial FiO_2_ and ventilator settings, bronchopulmonary dysplasia (defined as oxygen requirement at 36 weeks’ PMA), culture proven sepsis episodes. Lastly, NDI follow-up assessments at 2 years will include death or NDI as assessed at 22–26 months, death or hearing loss at 22–26 months and, death or cerebral palsy at 22–26 months.

### Study Assessments

Blood samples for the study will be obtained by the bedside nurse with clinically indicated routine blood draws. The first study blood sample (0.5 mL) will be obtained the morning after the maximum lipid dose is initiated (1.5 g/kg/d for the reduced lipid group; 3.0 g/kg/d for the usual lipid group). Blood samples 2–6 (each 0.25 mL) will be obtained on subsequent mornings through day 14 after birth. Total study blood volume will not exceed 2 mL (Table 3).

Whole blood will be collected in red-top microtainer (BD Microtainer, 2 mL), wrapped in aluminum foil, and placed in a photo-protective, amber-colored plastic bag at room temperature. Blood samples will be centrifuged within 12 hours of collection using *4G* for 10 minutes to extract serum which will be frozen at −80 degrees Celsius until samples are shipped in batch to Fluoresprobe Sciences in San Diego, California (A.K., A.H.). The Kleinfeld laboratory uses a novel method to measure UB using a fluorescent probe which compares favorably to the glucose-oxidase peroxidase-based method currently only available in Japan.(26) The fluorescent probe method determines the equilibrium UB concentration directly in a single analysis and is insensitive to substances that interfere with the peroxidase-based method, and is currently only available in research laboratories in the United States.(27)

Each whole blood sample measurements will include concentrations of UB, albumin, and total profiles for uFFA. The first sample will have additional individual profiles for uFFA. These results will not be available to the bedside clinician and will not affect direct patient care. These will be linked with all of the following measurements from the same blood sample measured in the hospital laboratory: TSB, indirect bilirubin, direct bilirubin, albumin, triglyceride, glucose, and bicarbonate. Additionally, if a blood gas is obtained within 4 hours of this blood draw, the pH from that blood gas will be linked to the sample. Other data reflecting general status and severity of illness and/or potentially influencing UB levels will be abstracted from the medical record, including baseline variables such as sex, gestation, anthropometrics, multiple gestation, delivery information, Apgar scores, and SNAPPE-II scores.(28) Growth Z-scores will be calculated at birth, 28 days after birth, and 34 and 36 weeks’ PMA.(25)

### BAER Recording

BAERs will be performed by two investigators (L.H., E.R.) with virtual real-time supervision by an expert in BAER methods (S.S.) with every patient encounter. Investigators (L.H., E.R.) will receive on-site training in two visits, and (L.H.) will continue with intensive weekly didactic and waveform reading sessions. BAERs will be performed at 34–36 weeks PMA or earlier if discharged from the hospital at < 34 weeks’ PMA or later if needed because of electrical interference associated with intensive care. BAERs will be assessed using carefully standardized methods.(29) Characterization and scoring of satisfactory waveforms will be confirmed by co-investigators (S.S., L.H.).

Each ear will be assessed individually with 80 dB normal hearing level, 100 microsecond (**msec**) clicks at a rate of 20.1 clicks per second, filtered from 100 Hz to 3 kHz and averaged for > 2000 repetitions. Prior to recording, impedance differences between ears of less than 10 kilo-Ohms will be ensured. The three rarefaction tracings will be averaged separately from the condensation, and also averaged with the condensation tracing all together. Rarefaction and condensation responses will be added together to cancel cochlear responses to distinguish from neural responses, necessary to critically diagnose auditory neuropathy. The most satisfactory waveform average will be used, with preference for right ear over left ear and rarefaction alone over combined rarefaction and condensation to maintain consistency in methods.

Because of the known physiologic development of BAER latencies associated with postnatal age, the PMA-adjusted wave V latency will be the primary predictor of interest. Other specific BAER measures will be studied including wave I latency, wave III latency, wave V latency, interwave interval I-III and III-V.

### NDI Follow-up Assessments

Follow-up appointments with children’s primary caretakers to review medical history, rehospitalization, and medical equipment use will occur with structured interviews. A standardized neurological exam and the Bayley Scales of Infant and Toddler Development, Fourth Edition (**BSID-IV**)(30) will be administered by certified examiners who maintain annual training to ensure interrater reliability at 22–26 months PMA. BSID-IV cognitive, language, and motor composite scores will be normalized to a mean of 100 (SD 15), with lower scores indicating more severe impairment. Motor impairment severity will be measured by the Gross Motor Function Classification System (**GMFCS**).(31) Cerebral palsy will be defined as mild with GMFCS level 1, moderate GMFCS level 2–3, and severe GMFCS level 4–5. Classification of severity of NDI is defined by a combination of BSID-IV scores and GMFCS levels.

### Data Collection, Management and Security

Data will be entered and stored in a secure McGovern Medical School REDCap database. Paper logs of data will be secured in a locked cabinet or room.

### Sample Size

We plan to assess BAERs in 60 infants in each treatment group and have more than 80% power at P < 0.05 to identify a difference between groups in wave V latency longer than 0.3 msec assuming a SD 0.56 msec. This sample size would also afford sufficient power to assess a moderate and clinically important difference in UB level in our center and allow for 92% attainment of wave V latency on BAERs.

### Statistical Analyses

Intention-to-treat analyses will be performed by a biostatistician (C.P.) blinded to group assignment. Generalized linear mixed models will be performed to analyze all outcomes and will include lipid dose and phototherapy groups (continuous and cycled) as covariates with random effects. Interaction will be assessed between lipid dose group and phototherapy group. Bayesian analyses will be performed in evaluating differences in treatment effects overall and in different patient subgroups of GA and birthweight strata. For the primary outcome and any other continuous outcome, a linear regression model will be used. Binary outcomes will be analyzed with a logistic regression and count data with a Poisson or negative binomial regression model. All priors will be neutral.

For binary outcomes, all priors will be centered at RR of 1.0 (indicative of no difference between treatment groups with a 95% prior interval of 0.3–3.0 (in the log RR scale a Normal Gaussian distribution with mean of 0 and variance of 0.50). A Normal (0,10^2^) prior will be used for the intercept term and Normal (0,1) for all other variables in the model. For the standard deviation of the center random effect, a half-Normal (0,1) prior will be used. Similar neutral informative priors(19) will be used for non-binary outcomes. We will report posterior medians and 95% credible intervals for group differences and relative risks. We will also report posterior probability of benefit/harm for each outcome. Similar analyses will be conducted for the cohort analyses. L.H., C.A., C.P., J.T. will have access to the final trial dataset.

### Data Safety Monitoring Board and Safety Reporting

The study statistician (C.P.) will conduct an interim analysis of mortality when half the planned sample size has completed the primary outcome, wave V latency. If there is a Bayesian posterior probability > 99% of mortality (assuming a neutral prior probability), the study statistician (C.P.), will notify DSMB members to decide whether to halt enrollment in the study. Together, the DSMB members will determine if there are important chance differences between treatment groups in baseline risk factors that are known to influence outcomes of ELBW infants (birthweight, GA, sex, singleton gestation, antenatal steroids, high ventilator settings) and that could account for differences in outcome.

### Dissemination of Results

Trial results will be communicated to the participants and the public via peer-reviewed publications. Additionally, we will also present our results to the Parents of Infants and Children with Kernicterus, a national organization that provides support and resources for families along with supporting research and treatments to improve the quality of life for those living with kernicterus. Based on their recommendation we will develop plans to create the best plan to disseminate our results to interested parents and the general public.

## Discussion

Originally developed for adult patients, pure soybean-based intravenous lipid emulsion has continued to be used as the primary lipid emulsion in the neonatal population for over 30 years without thorough investigation of the impact on BN despite more recent concerns.(7, 8, 32) This pilot study addresses whether a commonly used pure soybean-based intravenous lipid emulsion dosing regimen increases the risk BN toxicity in extremely preterm infants compared to administration of half the usual dose.

Rigorous pragmatic trials are needed to compare different lipid dosing while phototherapy dosing for the prevention of BN continues to evolve. This factorial design presents a unique opportunity to evaluate both therapies in the same high-risk population. Because an increase in the latency of BAER wave V latency is a sensitive marker of BN, we will evaluate whether wave V latency is decreased in the reduced lipid dose group compared to infants receiving the usual lipid dose irrespective of whether continuous or cycled phototherapy is used to manage the TSB. This study is limited to infants in our center enrolled in the larger NRN trial, and infants will be stratified by phototherapy group before randomization to receive usual or reduced dose lipid. Thus, this study employs a factorial design with four treatment groups with equal number of patients in each group. In the absence of an interaction among interventions as expected in our trial, conducting a factorial trial will allow the effect of both the lipid dose and mode of phototherapy on BAER wave V latency to be assessed in the same number of patients than to assess either intervention alone. For this trial, partly because of the minimal difference between the effect of cycled and continuous phototherapy findings on TSB, we do not anticipate a statistical interaction between lipid and phototherapy dosing. If an interaction is detected unexpectedly at the end of this trial, the analysis will report the degree of the interaction and provide a preliminary estimate of our primary outcome, and provide justification of need for a larger subsequent RCT.

BAERs can detect auditory pathway dysfunction and serve as a sensitive indicator of otherwise unrecognizable BN. Each of the waves reflect synchronized neuronal activity in the distal auditory nerve (wave I), the proximal auditory nerve (wave II), cochlear nucleus (wave III), superior olive (wave IV), and the lateral lemniscus (wave V), respectively. We chose wave V latency as the primary predictor for this study because it is rostral and thus affected when the highly sensitive cochlear nucleus (wave III) is abnormal, and it has been used in previous studies. Note that the BAER waveforms are present at 34–36 gestational week preterm birth, but latencies, i.e., the conduction time from the stimulus to the wave, shorten from birth to 2 years of age, maturing sooner for the earlier waves and later for each subsequent wave. BAER latencies are similar to adult latencies by 24 months after birth. Therefore, because of the known physiologic development of BAER latencies associated with postnatal age, and wave V latency will be the primary predictor of interest and will be adjusted for PMA. Interwave intervals I-III and III-V are used to measure the conduction times between the respective generators of those waves and peak-to-trough amplitude are available with no extra effort or testing, and will be analyzed as secondary variables.(33) Finally, we will record separately responses to rarefaction and condensation click stimuli to record cochlear microphonic responses, which is cases that have no replicable BAER responses can distinguish auditory dysfunction from BN from most other causes of no responses (e.g., genetic, ototoxic, malformation deafness, cochlear microphonic absent).

While BN with overt encephalopathy does occur in premature infants, it is rare in the United States and currently unpredictable.(34) Central auditory processing is affected by bilirubin toxicity and noted at TSB levels that were previously considered to be safe. Lasky et al(15) noted in infants 751–1000 g birthweight infants treated with conservative phototherapy compared to aggressive phototherapy had a 0.37 msec increased wave V latency (95% confidence interval, (CI)0.02–0.73), 0.39 msec increased wave III latency (95% CI 0.08–0.70), and 0.33 msec increased wave I latency (95% CI 0.01–0.65) between groups. Similar trends were seen in the 501–750 g birth weight group, although not significant. The chosen measure of prolongation of wave V latency by ≥ 0.3 msec was chosen based on these findings.(15)

This trial will provide further data to improve our understanding of the relationship of UB to BAERs and NDI. We expect difference between lipid dose groups in the first two weeks after birth in UB levels but not their BAERs at 34–36 weeks’ PMA or in rates of NDI at 2 years. This expectation is based in part on the absence of discernible BAER evidence of neurotoxicity with the usual lipid dose administered at our center in our recent pilot trial of cycled phototherapy,(19) and among infants with substantially higher TSB levels treated with conservative use of phototherapy in the prior NICHD NRN Trial.(15)

We hope our findings will help understand the complex relationship of bilirubin levels, risk factors, and clinical findings. If UB is higher and more prolonged BAER wave V latency is found in the usual lipid dose group in comparison to the reduced lipid dose group with either or both phototherapy groups will be worrisome, and support more cautious use of lipid emulsion use in the first two weeks after birth in ELBW infants. Values for UB and BAER wave V latency in the usual lipid dose group that are not greater than in the reduced lipid dose group in both phototherapy groups would be reassuring and support continued use of the usual lipid dose regimen to provide a somewhat greater caloric intake. If UB is higher with no discernible effect on wave V latency, the findings would suggest that the UB level were not high enough to cause BN with effects lasting to 34–36 weeks’ PMA. However, findings at 2 years would need to be scrutinized for evidence of NDI.

## Trial Status

The protocol version number NCT04584983 is on ClinicalTrials.gov at CMHH. The registration date was on October 14, 2020. The approximate date when recruitment will be completed on January 6, 2024.

## Figures and Tables

**Table 1:** Lipid Dosing for Treatment Groups.

Lipid Dosing Group	Day 1	Day 2	Day 3
**Days After Enrollment**	1	2	3
**Reduced Lipid Dose**	0.5 g/kg/d	1.0 g/kg/d	1.5 g/kg/d
**Usual Lipid Dose**	1 g/kg/d	2 g/kg/d	3 g/kg/d

**Table 2:** Participant Timeline.

Time point	Birth	Day 1	Day 2	Day 3	Day 4	Day 5	Day 6	Day 7	Day 8	Day 9	Day 10	Day 11	Day 12	Day 13	Day 14	Day 21	Day 28	34 weeks PMA	36 weeks PMA	Discharge	22–26 Months CGA
Eligibility	x	x	x																		
Informed Consent	x	x	x																		
Allocation		x	x																		
Randomization		x	x																		
Enrollment		x	x																		
Intervention		x	x	x	x	x	x	x	x	x	x	x	x	x	x						
Nutrition Intake	x	x	x	x	x	x	x	x	x	x	x	x	x	x	x	x	x	x	x		
Weight	x	x	x	x	x	x	x	x	x	x	x	x	x	x	x	x	x	x	x	x	x
Weight, Length, and Head Circumference Z Scores	x																x		x		
Blood Sample*					x	x	x	x	x	x											
Clinical Laboratory Data^#^					x	x	x	x	x	x											
BAER																		x	x	x	
Neonatal Clinical Outcomes																				x	
NDI Follow Up																					x

Brainstem Auditory Evoked Response (**BAER**); Corrected Gestational Age (**CGA**); Neurodevelopmental Impairment (**NDI**); Post-menstrual Age (**PMA**);

* Blood sampling is initiated the morning after the maximum lipid dose is started and occurs for 6 consecutive days. From these blood samples, the Kleinfeld laboratory will measure unbound bilirubin, albumin, total and full profile for unbound free fatty acids profile.

# These will be the clinically indicated blood sample measured in the hospital laboratory including, total serum bilirubin, indirect bilirubin, direct bilirubin, albumin, triglyceride, glucose, serum bicarbonate.

**Table 3:** Blood sample collection schedule.

Sample Number	Reduced Lipid Dose	Usual Lipid Dose	Age (days)	Blood Volume
*	0.5 g/kg/d	1.0 g/kg/d	0	0
*	1.0 g/kg/d	2.0 g/kg/d	1	0
*	1.5 g/kg/d	3.0 g/kg/d	2	0
1	*	*	3	0.50 mL
2	*	*	4	0.25 mL
3	*	*	5	0.25 mL
4	*	*	6	0.25 mL
5	*	*	7	0.25 mL
6	*	*	8	0.25 mL
			**Total Blood Volume**	<**2 mL**

## Data Availability

The dataset to be collected and analyzed for this study are available by contacting the corresponding author upon reasonable request.

## Abbreviations

BN: Bilirubin Neurotoxicity
UB: Unbound Bilirubin
BAER: Brainstem Auditory Evoked Response
RCT: Randomized Controlled Trial
GA: Gestational Age
FFA: Free Fatty Acids
uFFA: Unbound Free Fatty Acids
NDI: Neurodevelopmental Impairment
TSB: Total Serum Bilirubin
PMA: Post Menstrual Age
ELBW: Extremely Low Birth Weight
NICHD: National Institute of Child Health and Human Development
NRN: Neonatal Research Network
NICU: Neonatal Intensive Care Unit
SPIRIT: Standard Protocol Items: Recommendations for Interventional Trials
CMHH: Children’s Memorial Hermann Hospital
CGA: Corrected Gestational Age
Msec: Microsecond
BSID-IV: Bayley Scales of Infant and Toddler Development, Fourth Edition’
GMFCS: Gross Motor Function Classification System
CI: Confidence Interval
